# Prescribing patterns of glucagon-like peptide-1 receptor agonists in the Swedish capital region—a register-based cross-sectional study

**DOI:** 10.1007/s00228-025-03823-9

**Published:** 2025-03-13

**Authors:** Alice Fors, Tomas Forslund, Anders Sundström, Björn Wettermark

**Affiliations:** 1 Department of Pharmacy, Faculty of Pharmacy, Uppsala University, Box 580, 751 23 Uppsala, Sweden; 2 Department of Healthcare Development, Stockholm Region, Public Healthcare Services Committee, Stockholm, Sweden; 3 Academic Primary Health Care Centre, Stockholm Region, Stockholm, Sweden; 4 Department of Neurobiology, Care Sciences and Society, Division of Family Medicine and Primary Care, Karolinska Institutet, Solna, Sweden; 5 Division of Use and Information, Swedish Medical Products Agency, Uppsala, Sweden

**Keywords:** Prescribing patterns, Glucagon-like peptide-1 receptor agonists, Obesity, Diabetes, Sweden

## Abstract

**Purpose:**

Glucagon-like peptide-1 receptor agonists (GLP-1 RAs) have gained considerable media attention, but there is limited knowledge about those receiving the drugs. This study aimed to assess demographic characteristics and previous diagnoses in patients dispensed GLP-1 RAs in Region Stockholm, Sweden, between 2019 and 2023, with a focus on off-label prescribing.

**Methods:**

This was a register-based cross-sectional study including all inhabitants in Region Stockholm, Sweden, who were dispensed a GLP-1 RA between 2019 and 2023. Patient characteristics were assessed through record linkage with administrative healthcare data on demographics, healthcare consultations, diagnoses, and other dispensed drugs.

**Results:**

The prevalence proportion of GLP-1 RA dispensations in Region Stockholm increased from 4.7 patients/1000 inhabitants in 2019 to 17.5 patients/1000 inhabitants in 2023, and the incidence proportion from 1.8 patients/1000 inhabitants in 2019 to 7.4 patients/1000 inhabitants in 2023. GLP-1 RAs have become more common among a younger and female population, with women constituting 47% of incident patients in 2019 compared to 53% in 2023. The most common diagnosis shifted from type 2 diabetes mellitus (T2DM) (82% in 2019) to obesity (47% in 2023). During the same period, obesity without T2DM notably increased from 10 to 31%. Almost one-third (31%) of all patients dispensed the drugs in 2023 had no recorded diagnosis of either diabetes or obesity, compared to 8% in 2019.

**Conclusion:**

This study showed an increase in the dispensation of GLP-1 RA, with characteristics of patients changing towards a higher degree of off-label use. The effectiveness and safety of the increasing prescriptions warrant future studies.

## Introduction

In recent decades, several new blood glucose-lowering drugs have been introduced to the market. One of them is the glucagon-like peptide-1 receptor agonists (GLP-1 RAs), also called GLP-1 analogues, which today include seven substances: semaglutide, exenatide, liraglutide, lixisenatide, dulaglutide, albiglutide, and beinaglutide (see Table 2 in the Appendix). The GLP-1 RAs were originally developed for the treatment of type 2 diabetes mellitus (T2DM), but they soon showed promising effects in weight reduction [1, 2]. The substance liraglutide, under the brand name Saxenda®, was introduced to the market in 2015 as the first GLP-1 RA approved as a treatment complementary to exercise and diet for weight control in patients with high BMI (obese or overweight with weight-related comorbidities) [3]. The brand Wegovy®, containing the substance semaglutide, was the second brand to be introduced to the market indicated for obesity and overweight in 2022 [4]. Several randomized controlled studies have shown that GLP-1 RAs have a weight-lowering effect, but the magnitude varies between substances [5]. There is certain evidence that semaglutide is superior in reducing glycemia, body weight, BMI, waist circumference, and blood pressure [6]. Along with the positive effects on glycaemic control, the GLP-1 RAs have other beneficial effects on the cardiovascular system [5, 7], and semaglutide has been shown to reduce cardiovascular outcomes [8].

The prevalence of obesity and T2DM are increasing both globally [9, 10] and in Sweden [11, 12]. In 2021, 55% of adult men and 41% of adult women in Region Stockholm were estimated to be overweight or obese [13]. Moreover, a previous register study showed that 5% of all women and 7% of all men in the region were diagnosed with diabetes mellitus between 2007 and 2011 [14]. GLP-1 RAs offer new opportunities to manage T2DM, while also adding additional value in reducing the burden of obesity and overweight, as well as preventing cardiovascular disease. It is, however, important to acknowledge the other side of the coin: the GLP-1 RAs are associated with rapidly increasing expenditures [15], the use of falsified drugs [16, 17], and adverse events such as gastrointestinal adverse events which may be investigated more in the future along with the increased use of the GLP-1 RAs [18, 19]. Furthermore, due to huge global demand, in combination with limitations in the production capacity, there has been a global shortage of GLP-1 RAs throughout 2023 affecting the availability of semaglutide (Ozempic®) and liraglutide (Victoza®) in particular. As a consequence of the shortage situation, the Swedish Medical Products Agency published an open letter in November of 2023 urging healthcare professionals to only prescribe the drugs in question within the approved indication in order to ensure accessibility for diabetic patients [20–22].

In recent years, there has further been a rapid growth in online private clinics with a niche for weight-loss treatment, and the ground on which the physicians prescribe GLP-1 RAs has been criticized in the media [23–27]. Moreover, there have been reports of excessive off-label prescribing and misuse [28–33], and the GLP-1 RAs have become popular as a cosmetic weight loss drug by celebrities and are widely promoted on social media [34–39]. Therefore, studies assessing the quality of prescribing and characteristics of patients receiving GLP-1 RA are urgently needed.

This study aimed to assess demographic characteristics and previous diagnoses in patients dispensed GLP-1 RAs in Region Stockholm, Sweden, between 2019 and 2023, with a focus on off-label prescribing.

## Methods

This was a repeated cross-sectional study based on routinely collected healthcare data on dispensed drugs, recorded diagnoses, and demographic characteristics of patients dispensed at least one GLP-1 RA between 2019 and 2023.

### Setting

The study was conducted in the Swedish capital region, Region Stockholm, consisting of around 2.5 million inhabitants in 2023 [40].

Primary care constitutes the foundation of the Swedish healthcare system, though the organization differs between regions. Region Stockholm is characterized by its separation into smaller organizational units with the free establishment where payment to the providers is based on a combination of fee-for-service and capitation as a mode of governance. There are also large private and public specialist clinics providing care to many patients with diabetes and obesity to a greater extent than in other regions [41].

Relevant to the subject, Sweden has a “free prescription right” which means that a prescriber can, at his or her own discretion but judging it based on scientific evidence and proven experience, circumvent, e.g., the approved indication and prescribe a drug outside of the specifications in the Summary of Product Characteristics (SmPC), i.e., off-label. The basic principle is that the prescribing should be in line with the approval for the drug. However, in some cases, off-label use is acceptable, especially in therapeutic areas with few treatment alternatives [42]. The term “off-label drug use” refers to when a drug is used for medical purposes that deviate from the SmPC, as in the case of usage for an unapproved indication, population, or dosage [43, 44]. Notably, in this study, we only take unapproved indications into account, when referring to the term off-label, and use surrogate markers to identify this potential off-label use.

In Sweden, most prescription medications are included in the pharmaceutical benefits scheme, and the Dental and Pharmaceutical Benefits Agency of Sweden (TLV) is the authority that decides which medications are to be covered by the benefit [45]; thus, protecting the welfare state from spending money on drugs not deemed to be cost-effective, while protecting patients against high costs by utilizing a maximum yearly expense [46]. Two different types of subsidy can further cover a medication: a general subsidy, which includes its entire approved area of use, or a restricted subsidy, which restricts the subsidization to a particular area of use or group of patients [47]. All GLP-1 RAs other than Saxenda® and Wegovy® have the indication of T2DM and are subsidized only for patients with T2DM who first tried metformin, sulphonylureas or insulin, or where these are not appropriate. Notably, due to the ambiguities in the restriction text being pointed out by pharmacists and prescribers, TLV reconsidered their previous subsidy decision in March 2023 with the purpose of clarification. In the previous decision regarding Ozempic®, as an example, the benefit limitations were not stated as referring to the treatment of T2DM, even though this was the only medical condition mentioned in the investigation. However, in the new and clarified decision (2023), the limitations for subsidization of Ozempic® are as follows: “Subsidized only for patients with T2DM who first tried metformin, sulphonylureas or insulin, or where these are not appropriate” [48].

### Data source

In this study, information regarding the dispensed GLP-1 RA brand claimed benefit together with individual demographic and medical data were extracted from the health care data register VAL (Vårdanalysdatabaserna), a data warehouse governed by Region Stockholm which has previously been used in similar research [49, 50].

VAL itself is made up of several databases linkable with the unique identifiers of each patient, and these databases contain individual-level data on age, sex, dispensed prescription drugs, diagnoses, hospitalizations, healthcare consultations, economic transactions, as well as migration and death. The patient data originates from hospital-related care, specialized ambulatory care, and primary care. Previous research has shown that VAL has an almost complete coverage of all healthcare consultations in the region, except for a few private clinics operating under national contracts [51, 52].

Data on dispensed prescription drugs originate from all Swedish pharmacies. The selected data is complete with all drugs dispensed to inhabitants within Region Stockholm no matter where the prescriptions were issued or where in Sweden they were dispensed [53].

### Study population and covariates

The study population consisted of all inhabitants in Region Stockholm who had been dispensed a GLP-1 RA (Anatomical Therapeutic Chemical (ATC)-code: A10B, A10AE54, A10AE56) between January 2019 and December 2023. Both the substance and brand of the GLP-1 RAs were included since the subsidy is tied to the specific brand and not the substance. See Table 2 in the Appendix for details on all available brands with their corresponding ATC-codes.

**Fig. 1:**
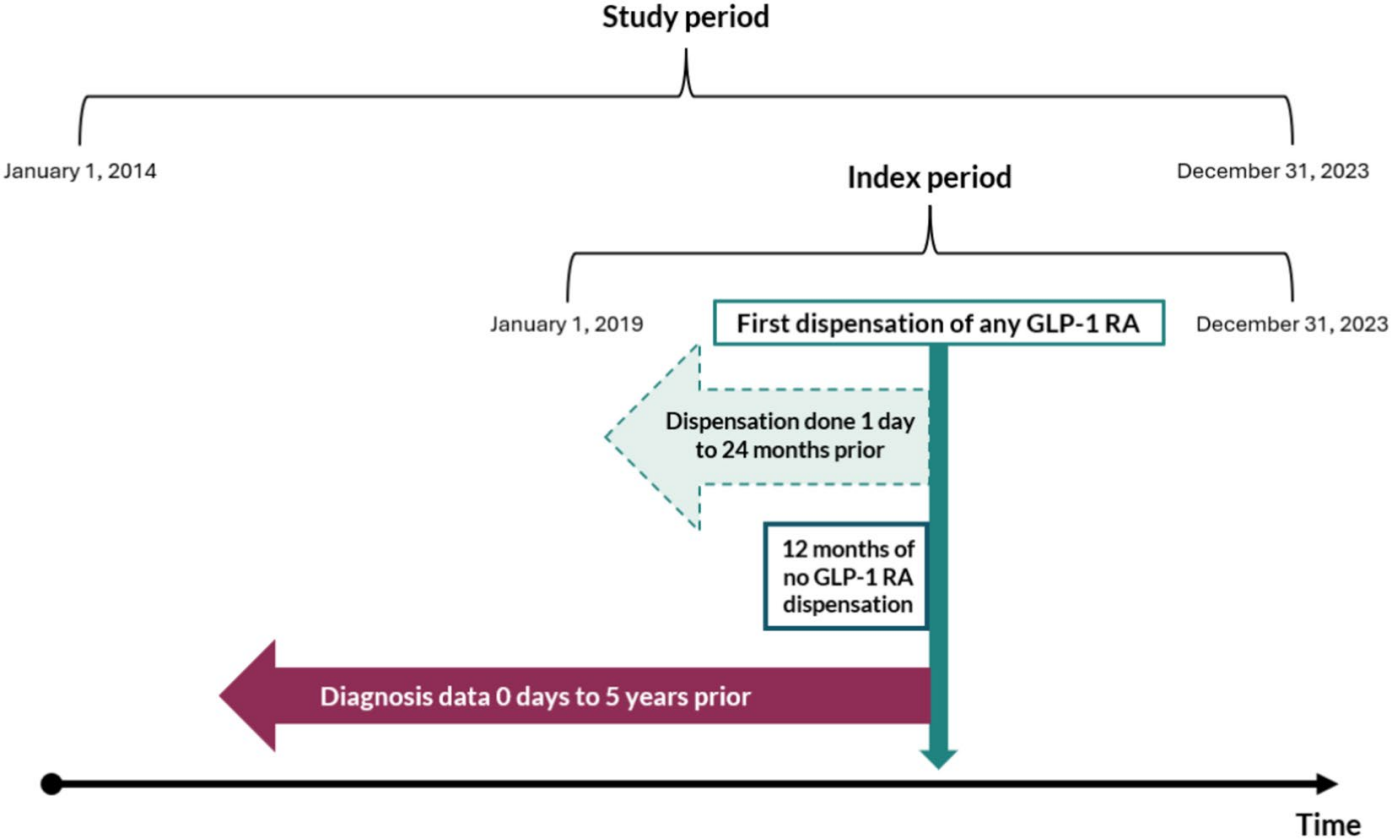
The study design explaining the GLP-1 RA dispensations within the index period, prior drug dispensation, and diagnosis within the whole study period

Diagnosis data were included within 5 years before the first dispensation of a GLP-1 RA (see Fig. 1). Selected diagnoses included T2DM/nonspecific DM, overweight/obesity, hypertension, heart failure, hyperlipidemia, and atherosclerotic CVD. Prior drug treatments included antihyperlipidemic agents, antidiabetic agents, antiplatelet agents, antihypertensives, and other anti-obesity agents dispensed up to 24 months before the first dispensation of any GLP-1 RA. See Table 3 in the Appendix for further details on ICD- and ATC-codes.

Patients who dispensed a GLP-1 RA for the first time within that same period had a run-in period of 12 months and were defined as incident patients, thus excluding patients who dispensed a GLP-1 RA in 2018 when assessing incidence in 2019. The 12-month wash-out period was selected according to Swedish prescription regulations where a prescription is valid for a maximum of 1 year. Incident patients were unique and were only included once. Prevalent patients were defined as unique individuals dispensed a GLP-1 RA within each year. The absence of a preexisting T2DM diagnosis, the absence of any previous antidiabetic drug, and prescribing outside of the benefit acted as surrogate markers for potential off-label prescribing of the GLP-1 RA brand with the indication of T2DM. The absence of a preexisting obesity diagnosis or any previous anti-obesity drug further acted as surrogate markers for potential off-label prescribing of the GLP-1 RA brand with the indication of obesity.

### Statistical analysis

Yearly prevalence and incidence were calculated as the number of prevalent and incident patients dispensed a GLP-1 RA, at least once during the year, divided by the Region’s yearly population [40]. The incidence was also calculated over the whole index period, 2019–2023.

The age of patients was divided into five age groups. Prevalent and incident GLP-1 RA patients were presented by sex for each year, whereas age group, substance, brand, previously recorded diagnoses, previous medications, and claimed benefit were presented for incident GLP-1 RA patients only.

Data from VAL was processed using SAS EG 8.2 software and analyzed in Microsoft Excel.

## Results

The proportion of the population dispensed a GLP-1 RA in the Stockholm Region, *i.e.,* the prevalence, increased between 2019 and 2023 from 4.7 patients/1000 inhabitants to 17.5 patients/1000 inhabitants (Table 1 (Panel A)). Women constituted a minority of the prevalent patients dispensed a GLP-1 RA between 2019 and 2022; however, this trend shifted in 2023 when women instead surpassed men.

**Table 1:** The prevalence and incidence, sex, and age distribution of patients dispensed a GLP-1 RA in Region Stockholm 2019–2023. Pat/TIN = patients/1000 inhabitants

	*2019*		*2020*		*2021*		*2022*		*2023*	
*Panel A: Prevalent patients*										
*Total prevalence (Pat/TIN)*	11112	4.7	14617	6.1	20231	8.4	28535	11.7	42962	17.5
*Sex*	***N***	**%**	***N***	**%**	***N***	**%**	***N***	**%**	***N***	**%**
*Men*	6208	55.9	8211	56.2	11192	55.3	15007	52.6	20507	47.7
*Women*	4904	44.1	6406	43.8	9039	44.7	13528	47.4	22455	52.3
*Panel B: Incident patients*										
*Total incidence (Pat/TIN)*	4256	1.8	5072	2.1	7201	3.0	10627	4.4	18170	7.4
*Sex*	***N***	**%**	***N***	**%**	***N***	**%**	***N***	**%**	***N***	**%**
*Men*	2251	52.9	2776	54.7	3796	52.7	4961	46.7	7310	40.2
*Women*	2005	47.1	2296	45.3	3405	47.3	5666	53.3	10860	59.8
*Age*	***N***	**%**	***N***	**%**	***N***	**%**	***N***	**%**	***N***	**%**
*<18*	5	0.1	9	0.2	23	0.3	40	0.4	92	0.5
*18–39*	419	9.8	488	9.6	675	9.4	1514	14.2	3725	20.5
*40–64*	2273	53.4	2693	53.1	3786	52.6	5796	54.5	10373	57.1
*65–79*	1398	32.8	1648	32.5	2339	32.5	2867	27.0	3546	19.5
*80+*	161	3.8	234	4.6	378	5.2	410	3.9	434	2.4

**Fig. 2:**
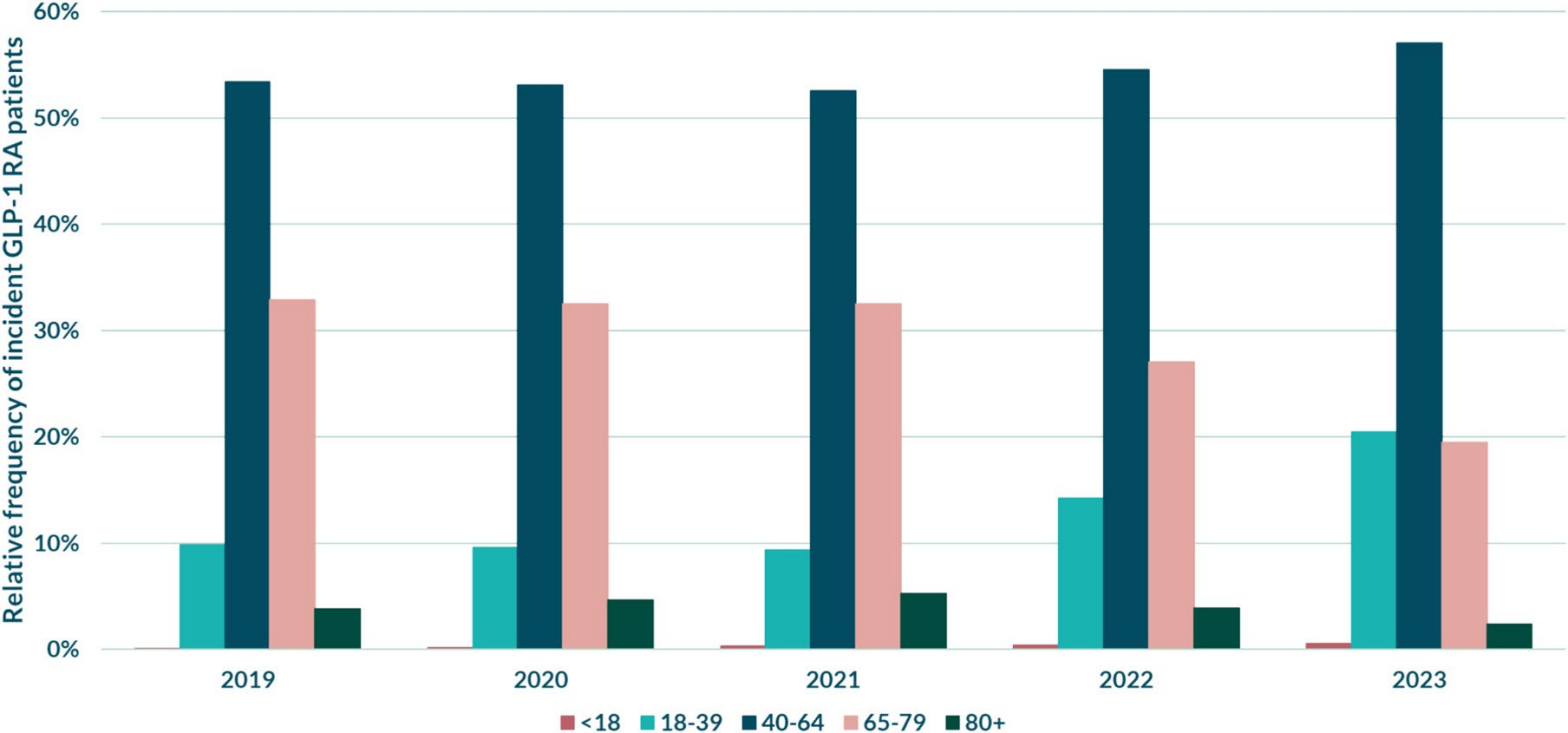
Incident patients distributed by age group and displayed in percent (%) between the years 2019 and 2023

As for the incident patients, an increase from 1.8 patients/1000 inhabitants in 2019 to 7.4 patients/1000 inhabitants in 2023 could be observed (Table 1 (Panel B)). An earlier shift in regard to sex can be seen among the incident patients, with women constituting the majority in both 2022 and 2023 (Table 1 (Panel B), Fig. 2). Another shift that can be observed among incident patients is that of the age distribution: the proportion of the three younger age groups increased after 2021, while the two older age groups decreased. The age group of 40–64 constituted the majority of incident patients over the entire period, and the least represented group overall was children and adolescents below the age of 18.

### Substance and brand

**Fig. 3:**
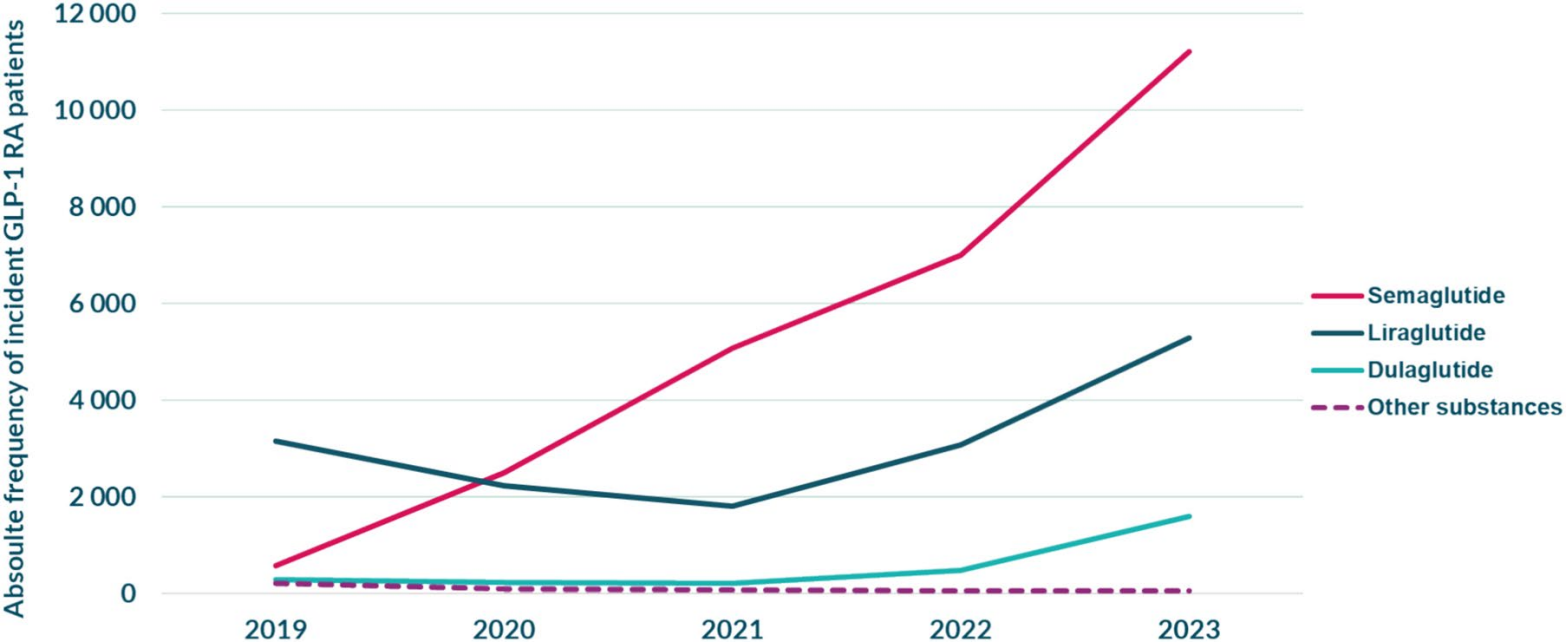
Number of incident patients dispensed different GLP-1 RA substances yearly. “Other” constitutes an amalgamation of lixisenatide+(A10AE54), liraglutide+(A10AE56), exenatide (A10BJ01), and lixisenatide (A10BJ03). See Table 4 in the Appendix for complete numbers

Liraglutide, followed by semaglutide, was the most frequently dispensed substance at initiations in 2019 (Fig. 3, Table 4 in the Appendix). However, this pattern shifted in 2020 when semaglutide became more frequent; a trend which continued, resulting in a close to twenty-fold increase 17.9% in incident semaglutide patients between 2019 and 2023. Dulaglutide was the third most frequent substance overall, and the remaining substances constituted a negligible share.

**Fig. 4:**
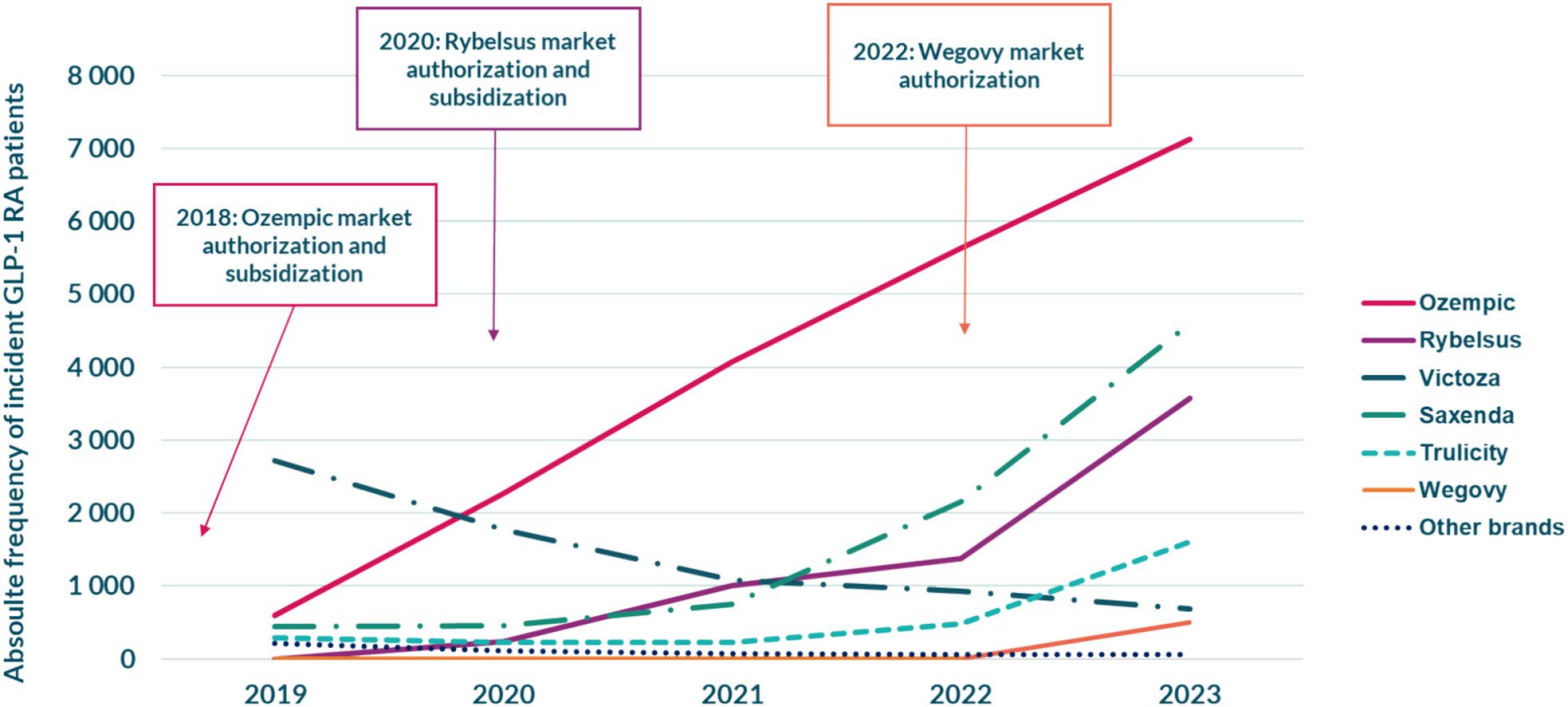
Number of incident patients dispensed different GLP-1 RA brands yearly. “Other brands” constitutes an amalgamation of Bydureon®, Byetta®, Lyxumia®, Suliqua®, and Xultophy®. Saxenda® and Wegovy Flex Touch®, notably, have the indication of obesity. The year of market authorization and subsidization are also included for brands approved after 2018. See Table 5 in the Appendix for complete numbers

As regards the different GLP-1 RA brands, Victoza® was the most dispensed brand of GLP-1 RAs among incident patients in 2019, with Ozempic® being the second most common, closely followed by Saxenda® (Fig. 4, Table 5 in the Appendix). Ozempic® increased substantially in 2020, surpassing Victoza®, and continued to make up the largest share in subsequent years, an increase of 182% between 2019 and 2023. The dispensation of Victoza® gradually decreased each year, whereas the dispensation of Saxenda® increased each year after 2020. Rybelsus® gained momentum in 2020 and increased every year thereafter, constituting the third most common brand in 2023, whereas Trulicity® started to increase in 2023. Wegovy® was not dispensed before the year 2023, and the dispensation of Bydureon®, Byetta®, Lyxumia®, Suliqua®, and Xultophy® constituted a negligible share overall.

### Previous diagnoses and medications

**Fig. 5:**
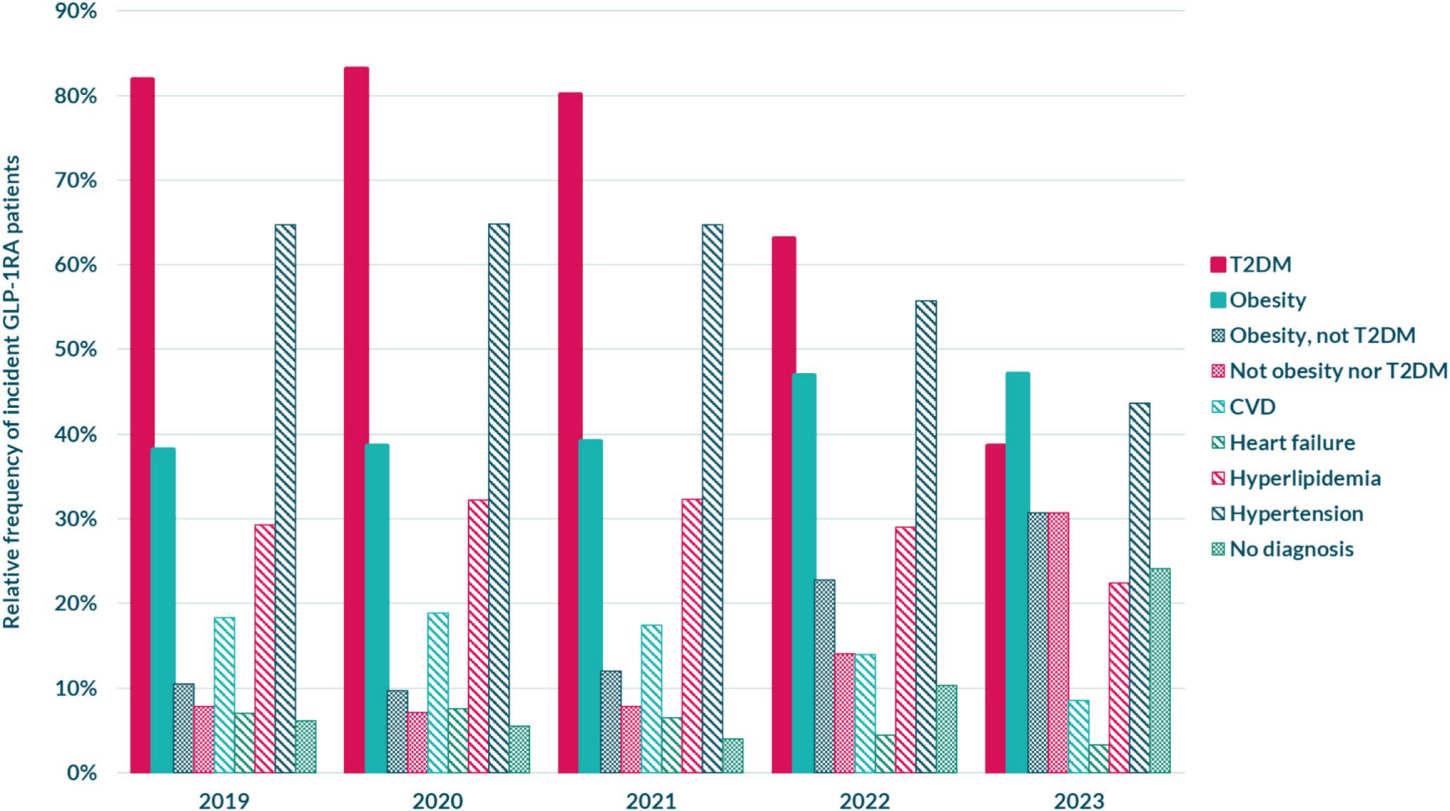
Diagnosis recorded within a 5-year period before incident patients claimed their first GLP 1 RA. Displayed in percent (%) for each year between 2019 and 2023. T2DM = type 2 diabetes mellitus + nonspecific diabetes mellitus. CVD = atherosclerotic cardiovascular disease. No diagnosis = none of the selected diagnoses. See Table 6 in the Appendix for complete numbers

Out of all 45,326 incident patients included within the entire period, 85.8% had at least one of the selected cardiovascular and metabolic diagnoses recorded in primary care or hospitals during 5 years before being dispensed their first GLP-1 RA, whereas 14.2% had not been given any of these diagnoses (Fig. 5, Table 6 in the Appendix).

T2DM was the most common diagnosis in incident patients overall (60.0%) and between the years 2019 and 2022; however, this pattern shifted in 2023 when both T2DM and the second most common diagnosis, hypertension, were surpassed by obesity (47.1%).

The proportion of incident users being diagnosed with obesity increased substantially between 2019 and 2023; obesity overall increased from 38.2% in 2019 to 47.1% in 2023, whereas obesity without T2DM increased from 10.5% in 2019 to 30.7% in 2023.

The most frequently dispensed medications to incident GLP-1 RA patients, dispensed at least once in the 24-month period before the first GLP-1 RAs, were antihypertensives (64.8%), followed by another antidiabetic drug (61.3%). Lipid-lowering medication was also frequent (47.6%), while antiplatelet (16.8%) and other anti-obesity drugs (5.9%) were frequent.

### Claimed pharmaceutical benefits

Out of all patients initiated on GLP-1 RAs between 2019 and 2023, nearly 80% had their first prescription dispensed within the pharmaceutical benefits. The relative proportion of incident patients dispensed these drugs outside the benefit increased by 59% between 2019 and 2023 (see Table 7 in the Appendix).

The majority of all prescriptions dispensed within the benefit were for Ozempic® (54.7%), and the majority outside the benefit was for Saxenda® (87.8%). Neither Saxenda® nor Wegovy® was ever dispensed within the benefit, whereas Bydureon®, Byetta®, Lyxumia®, Rybelsus®, and Suliqua® were always claimed to be dispensed within the benefit (see Table 8 in the Appendix).

Among incident patients dispensed a GLP-1 RA within the benefit, the most common previous diagnosis over the whole period was T2DM (74.1%). However, this changed in 2021 and onwards, and an overall decrease of 41% could be seen between 2019 and 2023. Within the same period, the dispensed prescriptions within the benefit to incident patients with a preexisting diagnosis of obesity without T2DM increased by 270% (4.0 to 14.6%), and 502% for patients with neither obesity nor T2DM (5.3 to 32.1%), and 515% for patients without any of the included diagnoses (4.2 to 25.7%) (see Table 9 in the Appendix).

For dispensations outside the benefit, both obesity (71.1%) and obesity without a T2DM diagnosis (68.1%) dominated overall and showed an increase of 31% and 76%, respectively, among incident GLP-1 RA patients between 2019 and 2023. During the same period, the diagnosis of T2DM decreased by 96% among patients initiated on a GLP-1 RA outside the benefit (see Table 9 in the Appendix).

## Discussion

This study assessed prescribing patterns of GLP-1 RAs in Region Stockholm, Sweden, between 2019 and 2023, with a focus on off-label prescribing. To the best of our knowledge, it is the first to investigate the prescribing patterns and off-label prescribing of GLP-1 RAs using data on diagnoses and dispensed prescriptions in an entire Swedish region. The results showed an exponential increase in both the prevalence and incidence of GLP-1 RA dispensation, with a channeling towards more women, younger persons, and those without a diagnosis of T2DM.

The rapidly increased prescribing of GLP-1 RAs is in line with several studies conducted across all continents [32, 54–60]. Our findings regarding the choice of GLP-1 RA substance and brand also mirror other studies [32, 33, 61]. These shifts in prescribing, regarding choice of substance and brand, could partially be explained by the recommendations issued by the Drug and Therapeutics Committee in Region Stockholm (Kloka listan) [62] and the year of market introductions of Ozempic® and Rybelsus®. Notably, more convenient formulations and administration, with Ozempic® offering a once-every-week injection [62] and Rybelsus® given orally [63], could also have contributed to the rise of these brands seen in this study. It should also be noted that during our study period, semaglutide was reported to exhibit both the highest weight-reducing effect [5, 18] and reduction of glycemia [6], which could be a contributory factor to the increase in the use of this substance in particular. Moreover, semaglutide and especially Ozempic® have gained extensive media coverage and praise on social media, where the public interest in Ozempic® has been reported to be the highest [28], which could have had a contributing effect on the high dispensation of this substance and brand [64, 65]. It is further possible that the increased demand for the semaglutide Ozempic®, resulting in a succeeding shortage situation in 2023 mostly affecting sales of Ozempic® and Victoza® as a result [20], could be a contributory factor to the rise of other substances and brands as a possible substitute when the low supply could not meet the high market demand.

A shift regarding previously registered diagnoses was further observed after 2021, when the proportion of both obesity and a lack of a registered T2DM diagnosis started to increase. This could partially be due to the high prevalence of obesity in Region Stockholm [13] and Sweden [11, 66], wherein a higher BMI has been reported to be significantly associated with a higher prescribing of GLP-1 RAs [67]. The increasing use of GLP-1 RAs could partially be driven by the large unmet demand for effective/efficient treatment in the obese population. The result could further be an indication of a substantial and rising off-label prescribing of GLP-1 RAs, something which aligns with previous indications of an increasing off-label prescribing and misuse of GLP-1 RAs [28–31, 60], together with a media picture of substantial misuse which has been reported in recent years [23, 26, 27, 34, 36, 68]. One of the driving factors behind this increase could be the ideal of thinness, which is further manifested through trends within social media [28, 69, 70], where both influencers and celebrities have praised and marketed GLP-1 RAs [37–39]. It should further be noted that there are examples of digital private clinics with a niche for weight-loss treatment that started in 2021 [71], and these most probably affected the rise of GLP-1 RAs observed after 2021.

The result further showed that the majority of patients who were dispensed their first GLP-1 RA received the drug within the pharmaceutical benefit, although the percentage of dispensations outside the benefit had increased. The relative increase in diagnoses other than T2DM seen among incident patients dispensed within the benefit could in turn indicate an increased off-label prescribing being done within the benefit. This could further point towards an increasing error in handling the benefits system, something which has previously been noticed by the Swedish pharmaceutical benefits agency TLV, resulting in a clarification regarding the subsidization limitations in 2023 [48]. It should, however, be noted that the result of this study only takes the first dispensation into account when referring to incident patients. Therefore, it is possible that later dispensations could have been done both within and outside of the benefit, something that has also been reported by the Swedish National Board of Health and Welfare [33].

The primary strength of this study was the high quality of the VAL database which covers all publicly funded healthcare within Region Stockholm, including primary care. It further covers all dispensed prescription drugs for the entire population in Region Stockholm, no matter where they are dispensed in Sweden, or if they were prescribed by a private health-care provider not funded by the region. Since the data extracted from the VAL databases was individual-level data, it was also possible to link the registers, thus connecting preexisting diagnosis and treatment to individual patients dispensed a GLP-1 RA. We also acknowledge that the study has some limitations. While we used a 12-month period to identify incident patients, it is possible that some people were not true incident patients but had a longer interruption in treatment. Diagnosis data were not available for some private healthcare providers on national contracts and online doctors not funded by the region, or care given by publicly funded digital healthcare providers based in other Swedish regions; hence, we cannot exclude the possibility that some patients have been given a diagnosis not included within this study. It is also possible that the patient obtained a diagnosis after being dispensed the drug, or before the 5 years included for preexisting diagnoses in this study design. Our results can therefore only reflect upon a potential increase in off-label use and not determine the definitive degree of off-label use. Another aspect to take into consideration is that we do not know what takes place at the pharmacy; it is possible that claimed benefits could have been changed during the dispensation at the pharmacy, in addition to the fact that the benefit could have been changed by the prescriber over time. It should further be noted that this study refers to off-label prescribing in the sense of prescriptions issued outside of the indication. However, the concept of off-label use is broader than what regards indication. Hence, this study cannot be seen as a complete investigation of all off-label use of GLP-1 RAs.

Despite these limitations, the study contributes new insight into the understanding of the GLP-1 RAs prescribing patterns. It may thus lay a basis for further studies using electronic health records or patient-reported data with the purpose of understanding who the potential off-label patients are and to what degree they benefit from treatment.

In conclusion, the results of this study show an increase in the dispensation of GLP-1 RAs as well as a shift in patient characteristics and choice of substance and brand. The result further indicates a higher degree of off-label use regarding indication and possible misuse of the subsidization system. However, future studies using more detailed clinical data need to be conducted to fully grasp the prescribing patterns and potential misuse of the subsidies.

## Data Availability

The pseudonymized patient-level data collected from regional registers are not allowed to share publicly due to confidentiality reasons; however, upon reasonable request, additional analyses can be conducted after contact with the corresponding author.

## Appendix

**Table 2:** List of all GLP-1 Ras, including information regarding their respective indication, approval, withdrawal, and reimbursement. It should be noted that EMA approved Wegovy® in January 2022; however, it has not been launched in Sweden yet and is only available through parallel import [4, 72, 73]. The indication of T2DM refers to complementary treatment to exercise and diet in patients with inadequately treated T2DM, which can be given either as an adjunct to existing treatment or, if the primary drug of choice (metformin) is considered inappropriate due to contraindications or intolerance, monotherapy can be an alternative [74, 75]. The indication of obesity/overweight here refers to complementary treatment to exercise and diet for weight control in patients with a high BMI (obese or overweight with weight-related comorbidities) [3, 4, 76, 77]

*Brand®*	*Substance*	*Indication*	*Authorization*	*Subsidized*
*Bydureon* [78, 79]	Exenatide	T2DM	17/06/2011	24/02/2012
*Byetta* [80, 81]	Exenatide	T2DM	20/11/2006	01/03/2010
*Saxenda* [3, 82]	Liraglutide	Obesity/overweight	23/03/2015	Not subsidized
*Victoza* [83, 84]	Liraglutide	T2DM	30/06/2009	27/01/2010
*Xultophy* [85, 86]	Liraglutide, insulin degludec	T2DM	18/09/2014	26/05/2015
*Suliqua* [87, 88]	Lixisenatide, insulin glargine	T2DM	11/01/2017	27/10/2017
*Lyxumia* [89, 90]	Lixisenatide	T2DM	31/01/2013	25/02/2015
*Eperzan* [91]	Albiglutide	T2DM	20/03/2014–9/10/2018	N/A
*Trulicity* [92, 93]	Dulaglutide	T2DM	21/11/2014	23/05/2015
*Ozempic* [74, 94]	Semaglutide	T2DM	08/02/2018	26/10/2018
*Rybelsus* [95, 96]	Semaglutide	T2DM	03/04/2020	28/08/2020
*Wegovy* [4, 77]	Semaglutide	Obesity/overweight	06/01/2022	Not subsidized
*N/A*	Beinaglutide	N/A	N/A	N/A

**Table 3:** Selected previously recorded diagnoses and prior medications with respective ICD- and ATC-codes

*Previously recorded diagnosis*	*ICD-code*
*T2DM/nonspecific DM*	*E11, E13, E14*
*Overweight/obesity*	*E66*
*Hypertension*	*I10-I13, I15*
*Heart failure*	*I50, I130, I132*
*Hyperlipidaemia*	*E78*
*Atherosclerotic CVD*	*I20-I25, I70, I739, I63-I64, I693, G450-G453, G455-G459, G46, Z866A, Z867C*
Prior *medication*	ATC-code
*Antihyperlipidemic agents*	*C10*
*Antidiabetic agents*	*A10 (excluding A10BJ)*
*Antiplatelet agents*	*B01AC*
*Antihypertensives*	*C03, C07-C09*
*Anti-obesity agents*	*A08AB01, A08AA62*

**Table 4:** Incident patients dispersed by substance displayed over time in absolute frequency (*N*) and percent (%). Lixisenatide+ = lixisenatide and insulin glargine as a fixed combination (A10AE54), Liraglutide+ = liraglutide and insulin degludec as a fixed combination (A10AE56). Dulaglutide (A10BJ05), exenatide (A10BJ01), liraglutide (A10BJ02), lixisenatide (A10BJ03), and semaglutide (A10BJ06)

*Substance*	*2019*		*2020*		*2021*		*2022*		*2023*		*Total*	
	*N*	%	*N*	%	*N*	%	*N*	%	*N*	%	*N*	%
*Dulaglutide*	294	6.9	229	4.5	223	3.1	485	4.6	1606	8.8	2837	6.3
*Exenatide*	7	0.2	4	0.1	1	0.01	1	0.01	0	0.0	13	0.03
*Liraglutide*	3152	74.1	2234	44.0	1822	25.3	3077	29.0	5287	29.1	15,572	34.4
*Liraglutide* ^*+*^	119	2.8	83	1.6	62	0.9	48	0.5	50	0.3	362	0.8
*Lixisenatide*	3	0.1	0	0.0	0	0.0	0	0.0	4	0.0	7	0.02
*Lixisenatide* ^*+*^	88	2.1	21	0.4	14	0.2	13	0.1	11	0.1	147	0.3
*Semaglutide*	593	13.9	2501	49.3	5079	70.5	7003	65.9	11,212	61.7	26,388	58.2
*All*	4256	100	5072	100	7201	100	10,627	100	18,170	100	45,326	100

**Table 5:** Incident patients distributed by brand displayed over time in frequency (*N*) and percent (%)

*Brand®*	*2019*		*2020*		*2021*		*2022*		*2023*		*Total*	
	*N*	%	*N*	%	*N*	%	*N*	%	*N*	%	*N*	%
*Bydureon*	5	0.1	3	0.1	1	0.01	0	0.0	0	0.0	9	0.02
*Byetta*	2	0.05	1	0.02	0	0.0	1	0.01	0	0.0	4	0.01
*Lyxumia*	3	0.1	0	0.0	0	0.0	0	0.0	4	0.2	7	0.2
*Ozempic*	593	13.9	2265	44.7	4075	56.6	5625	52.9	7129	39.2	19,687	43.4
*Rybelsus*	0	0.0	236	4.7	1004	13.9	1378	13.0	3577	19.7	6195	13.7
*Saxenda*	438	10.3	461	9.1	746	10.4	2155	20.3	4596	25.3	8396	18.5
*Suliqua*	88	2.1	21	0.4	14	0.2	13	0.1	11	0.1	147	0.3
*Trulicity*	294	6.9	229	4.5	223	3.1	485	4.6	1606	8.8	2837	6.3
*Victoza*	2714	63.8	1773	35.0	1076	14.9	922	8.7	691	3.8	7176	15.8
*Wegovy*	0	0.0	0	0.0	0	0.0	0	0.0	506	2.8	506	1.1
*Xultophy*	119	2.8	83	1.6	62	0.9	48	0.5	50	0.3	362	0.8
*All*	4256	100	5072	100	7201	100	10,627	100	18,170	100	45,326	100

**Table 6:** Diagnosis given within a 0-day to 5-year period before incident patients have been dispensed a GLP-1 RA within the index period. Displayed in frequency (*N*) and percent (%) for each year between 2019 and 2023 and as a total of the whole period

*Diagnosis*	*2019*		*2020*		*2021*		*2022*		*2023*		*Total*	
	*N*	%	*N*	%	*N*	%	*N*	%	*N*	%	*N*	%
*T2DM*	3488	82.0	4219	83.2	5771	80.1	6710	63.1	7021	38.6	27,209	60.0
*Obesity*	1626	38.2	1961	38.7	2822	39.2	4988	46.9	8557	47.1	19,954	44.0
*Obesity, not T2DM*	446	10.5	491	9.7	866	12.0	2419	22.8	5576	30.7	9798	21.6
*Not obesity nor T2DM*	332	7.8	362	7.1	564	7.8	1498	14.1	5573	30.7	8319	18.4
*CVD*	781	18.4	957	18.9	1259	17.5	1483	14.0	1543	8.5	6023	13.3
*Heart failure*	298	7.0	384	7.6	466	6.5	472	4.4	588	3.2	2208	4.9
*Hyperlipidaemia*	1247	29.3	1636	32.3	2326	32.3	3085	29.0	4076	22.4	12,370	27.3
*Hypertension*	2753	64.7	3289	64.8	4660	64.7	5919	55.7	7933	43.7	24,554	54.2
*No diagnosis*	262	6.2	279	5.5	429	4.0	1092	10.3	4378	24.1	6440	14.2

**Table 7:** Claimed benefit for incident patients dispensed GLP-1 RAs between 2019 and 2023, displayed over time in frequency (*N*) and percent (%)

*Benefit*	*2019*		*2020*		*2021*		*2022*		*2023*		*Total*	
	*N*	%	*N*	%	*N*	%	*N*	%	*N*	%	*N*	%
*Within benefit*	3493	82.1	4425	87.2	6439	89.4	8403	79.1	13,003	71.6	35,763	78.9
*Outside benefit*	763	17.9	647	12.8	762	10.6	2224	20.9	5167	28.4	9563	21.1
*Total*	4256	100.0	5072	100.0	7201	100.0	10,627	100.0	18,170	100.0	45,326	100.0

**Table 8:** Claimed benefit for incident patients dispensed different GLP-1 RA brands between 2019 and 2023, displayed in frequency (*N*) and percent (%)

*Brand®*	*Within benefit*		*Outside benefit*		*Total*	
	*N*	%	*N*	%	*N*	%
*Bydureon*	9	0.03	0	0.0	9	0.02
*Byetta*	4	0.01	0	0.0	4	0.01
*Lyxumia*	7	0.02	0	0.0	7	0.02
*Ozempic*	19,545	54.7	142	1.5	19,687	43.4
*Rybelsus*	6195	17.3	0	0.0	6195	13.7
*Saxenda*	0	0.0	8396	87.8	8396	18.5
*Suliqua*	147	0.4	0	0.0	147	0.3
*Trulicity*	2784	7.8	53	0.6	2837	6.3
*Victoza*	6713	18.8	463	4.8	7176	15.8
*Wegovy*	0	0.0	506	5.3	506	1.1
*Xultophy*	359	1.0	3	0.03	362	0.8
*All*	35,763	100.0	9563	100.0	45,326	100.0

**Table 9:** Diagnosis given within a 5-year period before incident patients have been dispensed a GLP-1 RA claimed to be within and outside of the benefit. Displayed in frequency (*N*) and percent (%) for each year between 2019 and 2023 and as a total of the whole period

Diagnosis	2019		2020		2021		2022		2023		Total	
	*N*	%	*N*	%	*N*	%	*N*	%	*N*	%	*N*	%
Within benefit												
T2DM	3169	90.7	4040	91.3	5745	89.2	6615	78.7	6936	53.3	26,505	74.1
Obesity	1205	34.5	1572	35.5	2245	34.9	3309	39.4	4821	37.1	13,152	36.8
Obesity, not T2DM	138	4.0	159	3.6	301	4.7	792	9.4	1899	14.6	3289	9.2
Not obesity nor T2DM	186	5.3	226	5.1	393	6.1	996	11.9	4168	32.1	5969	16.7
CVD	713	20.4	912	20.6	1239	19.2	1413	16.8	1393	10.7	5670	15.9
Heart failure	272	7.8	363	8.2	454	7.1	451	5.4	540	4.2	2080	5.8
Hyperlipidaemia	1123	32.2	1539	34.8	2254	35.0	2849	33.9	3521	27.1	11,286	31.6
Hypertension	2449	70.1	3049	68.9	4447	69.1	5337	63.5	6473	49.8	21,755	60.8
No diagnosis	146	4.2	173	3.9	300	4.7	696	8.3	3344	25.7	4659	13.0
Total patients within benefit	3493	100.0	4425	100.0	6439	100.0	8403	100.0	13,003	100.0	35,763	100.0
Outside benefit												
T2DM	319	41.8	179	27.7	26	3.4	95	4.3	85	1.6	704	7.4
Obesity	421	55.2	389	60.1	577	75.7	1679	75.5	3736	72.3	6802	71.1
Obesity, not T2DM	308	40.4	332	51.3	565	74.1	1627	73.2	3677	71.2	6509	68.1
Not obesity nor T2DM	136	17.8	136	21.0	171	22.4	502	22.6	1405	27.2	2350	24.6
CVD	68	8.9	45	7.0	20	2.6	70	3.1	150	2.9	353	3.7
Heart failure	26	3.4	21	3.2	12	1.6	21	0.9	48	0.9	128	1.3
Hyperlipidaemia	124	16.3	97	15.0	72	9.4	236	10.6	555	10.7	1084	11.3
Hypertension	68	8.9	45	7.0	20	2.6	70	3.1	150	2.9	353	3.7
No diagnosis	116	15.2	106	16.4	129	16.9	396	17.8	1034	20.0	1781	18.6
Total patients outside benefit	763	100.0	647	100.0	762	100.0	2224	100.0	5167	100.0	9563	100.0
